# Simultaneous differential detection of *Chlamydophila abortus, Chlamydophila pecorum *and *Coxiella burnetii *from aborted ruminant's clinical samples using multiplex PCR

**DOI:** 10.1186/1471-2180-9-130

**Published:** 2009-07-01

**Authors:** Mustapha Berri, Abdessalem Rekiki, Karim Sidi Boumedine, Annie Rodolakis

**Affiliations:** 1 Institut National de la Recherche Agronomique (INRA), UR1282, Infectiologie Animale et Santé Publique (IASP), F-37380 Nouzilly, France; 2 Institut de la Recherche Vétérinaire de Tunisie, La Rabta, Tunis 1006, Tunisia

## Abstract

**Background:**

Chlamydiosis and Q fever, two zoonosis, are important causes of ruminants' abortion around the world. They are caused respectively by strictly intracellular and Gram negative bacterium *Chlamydophila abortus (Cp. abortus*) and *Coxiella burnetii (C. burnetii). Chlamydophila pecorum (Cp. pecorum) *is commonly isolated from the digestive tract of clinically inconspicuous ruminants but the abortive and zoonotic impact of this bacterium is still unknown because *Cp. pecorum *is rarely suspected in abortion cases of small ruminants. We have developed a multiplex PCR (m-PCR) for rapid simultaneous differential detection of *Cp. abortus*, *Cp. pecorum *and *C. burnetii *in clinical samples taken from infected animals.

**Results:**

Specific PCR primers were designed and a sensitive and specific m-PCR was developed to detect simultaneously, in one tube reaction, three specific fragments of 821, 526 and 687-bp long for *Cp. abortus, Cp. pecorum *and *C*. *burnetii *respectively. This m-PCR assay was performed on 253 clinical samples taken from infected ruminant's flocks that have showed problems of abortion diseases. Thus, 67 samples were infected by either one of the three pathogens: 16 (13 vaginal swabs and 3 placentas) were positive for *Cp. abortus*, 2 were positive for *Cp. pecorum *(1 vaginal swab and 1 placenta) and 49 samples (33 vaginal swabs, 11 raw milks, 4 faeces and 1 placenta) were positive for *C. burnetii*. Two vaginal swabs were m-PCR positive of both *Cp. abortus *and *C. burnetii *and none of the tested samples was shown to be infected simultaneously with the three pathogens.

**Conclusion:**

We have successfully developed a rapid multiplex PCR that can detect and differentiate *Cp. abortus*, *Cp. pecorum *and *C. burnetii; *with a good sensitivity and specificity. The diagnosis of chlamydiosis and Q fever may be greatly simplified and performed at low cost. In addition, the improvement in diagnostic techniques will enhance our knowledge regarding the prevalence and the pathogenetic significance of Q fever and chlamydiosis.

## Background

Chlamydiosis and Q fever, two zoonosis, are widely distributed around the world. Their importance is related not only to the economic losses in animal production, but also to risks posed to humans [1,2]. They are caused respectively by strictly intracellular and Gram negative bacterium *Chlamydophila *and *Coxiella burnetii*. Although *C. burnetii *and *Chlamydophila *belong to phylogenetically unrelated species [3], they show some similarities in their interaction with the host and pathogenesis of the infection [4]. Chlamydiaceae family is composed of nine species recognized within the two genera of *Chlamydia *and *Chlamydophila *[5] which are associated with a large variety of diseases in animals and humans including abortion, pneumonia, gastroenteritis, encephalomyelitis, conjunctivitis, arthritis and sexually transmitted diseases [6]. The reservoir is large and includes many wild and domestic mammals but domestic ruminants such as sheep, cattle and goat represent the most frequent source of human infection. Two species of the genus *Chlamydophila *cause diseases in ruminants, *Chlamydophila abortus *(formerly *Chlamydia psittaci *serotype 1) and *Chlamydophila pecorum (*formerly *Chlamydia pecorum*). *Cp. abortus *has an affinity for placenta tissue and is an important cause of reproductive disorders such as abortion or premature births in pregnant sheep, goat and is also hazardous for pregnant women [1,6]. Although *Cp. pecorum *is commonly isolated from the digestive tract of clinically inconspicuous ruminants, this bacterium was recognized to be as a cause of fertility disorders, conjunctivitis, arthritis, mastitis, pulmonary inflammation, in sheep, goat and cattle [7-10]. Although the role of *Cp. abortus *and *C burnetii *as aetiological agents of abortion has been clearly established in humans and ruminants, the abortive and zoonotic impact of *Cp. pecorum *is still unknown. Nevertheless, *Cp. pecorum *involvement in small ruminants abortion cases has been previously reported, almost 20 years ago, in south of France [11]. Recently, during the course of collaboration studies between our laboratory and veterinary institutes of Morocco, Algeria and Tunisia, *Cp. pecorum *strains were isolated from abortion cases of goat [12] and sheep (unpublished data) suggesting that this bacterium might be involved in small ruminants abortion in North African countries.

Like chlamydiosis, the main reservoir of human Q fever is infected ruminants that shed *C. burnetii *into the environment during normal delivery or abortion through the amniotic fluids and the placenta as well as via faeces and milk [13,14]. The transmission of infections to humans is mainly due to the inhalation of contaminated aerosols, but may also occur following the consumption of raw milk and dairy products [15,16]. Furthermore, contaminated faecal samples and manure brought from a farms housing infected ruminants have been involved as sources of humans Q fever [17]. Improved diagnostic methods of *Chlamydia *and *Coxiella *detection is required to prevent both human and animal contamination. Chlamydiosis and Q fever diagnosis is usually established by bacterioscopic examination of stained placenta smears which are poorly sensitive and not specific. Isolation is also employed, but it is difficult, time consuming, hazardous, and the organism requires level 3 (P3) containment facilities for propagation. The simplest methods for detecting infected animals rely on the detection of *Coxiella *and *Chlamydia *antibodies in animal sera, such by immunofluorescence, ELISA and the complement fixation tests. These methods are presumptive and rely on time for antibody production to occur; thus, they are not early-detection methods. Furthermore, cross-reactivity between *C. burnetii and Chlamydia *strains in ELISA and immunoblot analysis was observed [18]. Molecular methods such as PCR have been developed for each individual pathogen and have demonstrated a high sensitivity and specificity [19-21]. A duplex PCR was recently developed to simultaneously detect *Cp. abortus *and *C. burnetii *in broad range of abortion products in cattle [22]. Thus, we decided to develop a rapid, economic, sensitive, and specific multiplex PCR (m-PCR) for simultaneous detection and differentiation of *Cp. abortus, Cp. pecorum *and *C. burnetii *in clinical samples of ruminants. The application of this improved PCR test will enable accurate, epidemiological and prevalence data of Chlamydiosis and Q fever, which in turn will lead to an increase the efficiency of animal production and reduction in zoonotic transmission to humans.

## Methods

### *Chlamydophila *and *Coxiella burnetii *strains

Twenty strains of *Cp*. *abortus*, 5 strains of *Cp. pecorum*, and 4 strains of *C. burnetii *including the reference strain *Cp*. *abortus *AB7, *Cp. pecorum *iB1 and *C. burnetii *Nine-Miles were used in this study. All these strains were isolated from ruminants except Nine Miles, which was isolated from ticks.

### Animals

In this study, a total of 11 sheep and goat flocks were investigated including seven flocks located in five different regions of Tunisia, 3 flocks located in two different regions of France (Touraine and Alpes-de-Hautes-Provence) and the flock belonging to the experimental unit of INRA Research Centre of Tours-Nouzilly (France) where Chlamydiosis and Q fever-related abortions were suspected. Q fever and Chlamydiosis serological responses were tested in each flock on 20 selected animals, including all females that aborted and some females that delivered normally using ELISA tests (Pourquier, Montpellier, France) and (CHEKIT^R^, Hoechst Roussel Vet, France) respectively following the manufacturer recommendations.

### Collection and clinical sample preparation

The samples used in this study are listed in Table 1. A total of 253 clinical samples were taken from all animals that aborted and among both ELISA positive and negative animals that delivered normally. Thus, 72 clinical samples were collected by the Institute of Veterinary Research of Tunisia and a total of 102 samples were obtained from a group of reproduction of 34 ewes belonging to the experimental unit of INRA Research Centre of Tours-Nouzilly (France). The French county veterinary laboratories of Touraine (VCL37) and of Alpes-de-Hautes-Provence (VCL04) collected 5 placentas and a total of 74 samples, respectively. The gestation statue of the sampled animals was recorded and all tested animals were identified and correlated with the serology result and the samples were analysed by PCR. DNA preparation and purification were performed following the protocol described by [23].

**Table 1:** Samples tested for m-PCR validation

Geographic locality	Animal's specie	Samples			
		Placentas	Vaginal swabs	Milks	Feces
		
**France**					
VCL 04	Ovine		15		
	Bovine			2	1
	Caprine		28	28	
					
Experimental Unit (INRA-Tours)	Ovine		34	34	34
VCL 37	Ovine	1			
	Bovine	1			
	Caprine	3			
					
**Tunisia**					
Institute of Veterinary Research	Ovine		71		
	Caprine		1		
					
**Total**		**5**	**149**	**64**	**35**

A total of 253 clinical samples including placentas, vaginal swab milk and feces were taken from ruminants flocks belonging to different geographic localities in France and in Tunisia. The gestation statue of the sampled animals was recorded and all tested animals were identified.

### PCR analysis

#### Primers

All primers used in this study were synthesized by Sigma-Genosys (Sigma Aldrich, Saint Quentin Fallavier, France). The name, sequence, target gene, the predicted amplified fragment, as well as the melting temperature are listed in Table 2. Primers pmp F and pmp 821R were designed from the four pmp gene sequences of *Cp. abortus *S26/3 strain [24]. RAPD-PCR analysis was used to investigate the molecular epidemiology of several isolates of *Chlamydophila *and, as shown, *Cp. pecorum *strains were distinguished from the others by the presence of 650-bp specific fragment in electrophoresis [25]. A set of CpcF and CpcR primers were designed based on the DNA sequencing of this fragment in order to obtain *Cp. pecorum *specific amplification product. Trans-1 and Trans-2 PCR primers were described previously and designed based on the transposon like repetitive region of *C. burnetii *[26].

**Table 2:** The targeted genes and PCR primers used for the detection and the differentiation of *Cp. abortus*, *Cp pecorum *and *C. burnetii*.

Target gene	Primers name	Primers sequence (5'-3')	Amplified fragment length (bp)	Melting temperature (°C)
pmp 90/91	pmp-F	CTCACCATTGTCTCAGGTGGA	821	64
	pmp-R821	ACCGTAATGGGTAGGAGGGGT		66.3

CPC	Cpc-F	TTCGACTTCGCTTCTTACGC	526	64.3
	Cpc-R	TGAAGACCGAGCAAACCACC		67.4

IS1111a	Trans-1	TATGTATCCACCGTAGCCAGT	687	67.5
	Trans-2	CCCAACAACACCTCCTTATTC		66

The name, the sequence, the target gene and the predicted amplified fragment, as well as the melting temperature are listed.

#### PCR conditions

Precautions were taken to use sterile reagents and conditions, and contamination of reactions by PCR product was avoided by strict separation of working areas and use of filter pipette tips. The optimal PCR conditions for *Cp. abortus, Cp. pecorum *or *C. burnetii *individual amplification were initially determined separately using serial dilutions of respective DNA solution. PCR reactions were carried out in a final volume of 25 μl containing 1× PCR buffer (Promega, Charbonnières-Les-Bains, France), 0.5 μM of each primer set, 200 μM of the four deoxynucleoside triphosphate (dATP, dGTP, dCTP, dTTP), 2 mM MgCl_2 _and 0.5 U of Taq polymerase (Promega, Charbonnières-Les-Bains, France). PCR reactions were performed in an automated DNA thermal cycler (Eppendorf, Le Pecq, France). After an initial denaturation period of 10 min at 94°C, reactions were subjected to 35 cycles of 30 sec at 94°C, 1 min at an annealing temperature of 63°C for *Cp. abortus*, 62°C for *Cp. pecorum *and 64°C for *C. burnetii*, then 72°C for 1 min with a final extension step at 72°C for 10 min.

#### m-PCR conditions

In order to simultaneously detect the three bacteria, the reactions were subsequently combined to develop a one-step reaction. Testing different combinations of the reaction mixture components allowed the performing an optimization of the multiplex PCR assay (m-PCR). A good intensity of the amplified fragment for each target DNA as well as the absence of unspecific bands were considered in selecting the optimal m-PCR conditions. Thus, the best results were obtained when the final concentration of the three primer sets, MgCl_2_, and Taq polymerase was increased respectively to 0.8 μM, 3 mM and to 1.5 U and the m-PCR was carried out in a final volume of 50 μl. The thermal cycler parameters of the m-PCR were similar to those of the individual PCR using 61°C as an optimal annealing temperature. Positive and negative control DNA samples were run in each experiment. PCR products were analyzed in 1.2% agarose gel electrophoresis, stained with ethidium bromide and visualised with ultraviolet transillumination. All PCR reactions assessing limits of detection or specificity were performed in duplicate.

### Sensitivity and specificity of the m-PCR

Sensitivity of the PCR assay was checked using serial fold dilutions of bacterial suspension of references strains AB7, iB1 and Nine-Miles at 10^7 ^bacteria per ml. Simulated positive samples were also obtained by adding 50 μl of bacterial suspension dilution to 50 μl of bacteria-free vaginal swab extract or milk sample. These preparations were then submitted to extraction procedures and to simplex and m-PCR as described above.

The specificity of the PCR was assessed on 20 strains of *Cp*. *abortus*, 5 strains of *Cp. pecorum *and, 4 strains of *C. burnetii *from our laboratory bacteria collection and on some isolates suspected to be present into tested clinical samples: *Brucella melitensis, Brucella abortus, Brucella suis, Escherichia coli, Bacillus cereus, Listeria monocytogenese, Salmonella abortus ovis, Salmonella Typhimurium, Staphylococcus aureus, Staphylococcus chromogenese, Staphylococcus hominis, Streptococcus dysgalactiae *and *Streptococcus ogalactiae, Mycobacterium avium, Legionella pneumophila*. In addition, RFLP-PCR analysis was carried out as a confirmatory test for the PCR reaction specificity. Thus, 10 μl of amplification products obtained from naturally infected clinical samples and those obtained from 10^2 ^genomic DNA templates of the reference strains AB7, IB 1, Nine Miles were subjected to 5 units of *AluI *restriction enzyme (Promega, Charbonnières-Les-Bains, France) in a 20 μl final volume for 3 hours at 37°C. The digested products were examined by using 2% agarose gel stained with ethidium bromide and viewed under UV illumination. In addition, PCR products amplified from clinical samples were purified with a QIAquick PCR purification Kit (Qiagen, Courtaboeuf, France) and directly sequenced with an ABI PRISM 310 genetic analyzer (Applied Biosystems).

### Isolation of *Chlamydophila *and *Coxiella *strains

Pathogen isolation was performed to confirm the presence of the involved bacteria, on 20-different PCR positive samples showing high ethidium bromide intensity on agarose gel. *Chlamydophila *strains isolation were performed using both plaque assays and blind passages on McCoy monolayer cell cultures [27]. Briefly, PCR positive samples for *Cp. abortus *or *Cp. pecorum *were first diluted to 1:10 and subsequently used in a plaque assay. Furthermore, 500 μl of this suspension was added to McCoy cell monolayers in 25 cm^2 ^flasks to perform the blind passage assay. The positive culture and plaque cloned *Chlamydophila *were then grown in specific pathogen-free eggs, the yolk sacs were harvested one week later and the bacteria were purified and stored at -80°C. *C. burnetii *strains were isolated by intraperitoneal inoculation of OFI mice then on embryonated hen eggs [28]. Briefly, 3 OF1 mice (8 weeks old) were inoculated with 0.2 mL of vaginal swab extract or milk sample tested positive in PCR. The mice were killed nine days post inoculation and the spleens were sampled and reinoculated into 6-days-old, specific pathogen-free embryonated hen eggs. The infected yolk sacs of dead and viable embryos were harvested between 8 and 10 days after inoculation, aliquoted and frozen at -80°C. Genomic DNA of isolated *chlamydophila *and *Coxiella *was prepared using a QIAmp DNA mini Kit (Qiagen, Courtaboeuf, France) following the manufacturer's recommendations and characterized using RFLP-PCR method of 16S–23S rRNA intergenic region [29].

## Results

### Initial set-up and optimization

**Figure 1:**
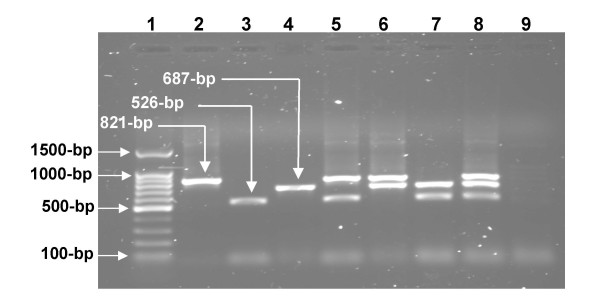
**Multiplex PCR amplification of *Cp. abortus*, *Cp. pecorum *and *C. burnetii *references strains individually, and in all possible combinations**. Lane 1: 100-bp ladder; lane 2: *Cp. abortus *AB7; lane 3: *Cp. pecorum *iB1; lane 4: *C. burnetii *Nine Miles; lane 5: *Cp. abortus *and *Cp. pecorum; *lane 6:*Cp. abortus *and *C. burnetii*; lane 7: *Cp. pecorum *and *C. burnetii*; lane 8: *Cp. abortus, Cp. pecorum *and *C. burnetii*; lane 9: Negative control without DNA. The sizes of the three different PCR products are shown on the left.

The primer sets pmpF/pmpR821, CpcF/CpcR and Trans-1/Trans-2 designed in this study, challenged simultaneously with DNA extracts of AB7, iB1 and Nine-Miles reference strains of *Cp. abortus, Cp. pecorum*, and *C. burnetii *resulted in a micro-organism-specific identification of the target sequence. The amplification conditions and master mixture components were optimized to amplify all DNA as singlet, in different combinations as duplexes or as triplex of three target sequences (Figure 1). With a primer concentration of 0.8 μM, 1.5 U of Taq polymerase, 3 mM of MgCl_2 _and an annealing temperature of 61°C, m-PCR produced simultaneously in one tube reaction, three specific fragments of 821, 526 and 687-bp long for *Cp. abortus, Cp. pecorum *and for *C*. *burnetii*, respectively. No m-PCR product was generated using water instead of target DNA (Figure 1)

### Sensitivity and specificity of PCR

**Figure 2:**
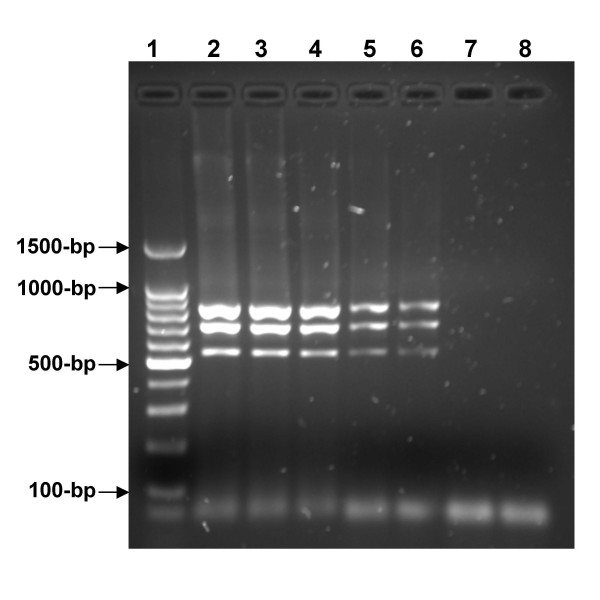
**Sensitivity of Multiplex PCR amplifying simultaneously *Cp. abortus *AB7, *Cp. pecorum *iB1 and *C. burnetii *Nine Miles reference strains**. Lane 1: 100-bp ladder; lane 2–7: variation of total genomic DNA amount isolated from the three bacteria (10^5^, 10^4^, 10^3^, 10^2^, 50 and 10 genome copies per PCR reaction); lane 8: Negative control without DNA.

**Figure 3:**
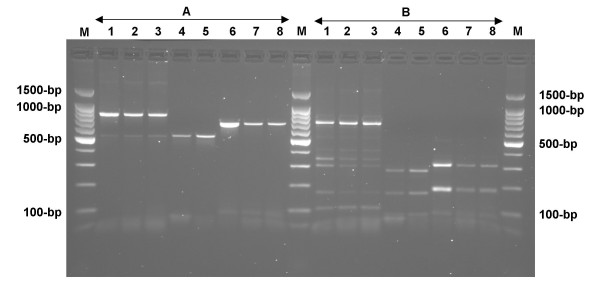
**Electrophoresis analysis of PCR products amplified using pmp/pmpR821, CpcF/CpcR or Trans-1/Trans-2 primers sets on either AB7, iB1, Nine Miles references strains or naturally infected biological samples (A) and their respective RFLP profiles after digestion with *AluI *(B)**. M: 100-bp ladder. Lane 1: *Cp. abortus *AB7; lanes 2 and 3: vaginal swab taken from two aborted ewes; lane 4: *Cp. pecorum *iB1; lane 5: vaginal swab taken from aborted ewe; lane 6: *C. burnetii *Nine Miles; lanes 7 and 8: Milk sample taken from two aborted goats.

m-PCR, as well as duplex or single PCR performed on reference strain (AB7, iB1 and Nine-Miles) purified DNA with the same primers, detected as little as 50 genome copies per PCR reaction (Figure 2). Experimental contamination of clinical samples with decreasing amounts of bacteria was performed for the evaluation of the detection threshold of the PCR technique. Both vaginal swab and milk samples did not interfere with m-PCR performance, since the same detection threshold was observed (data not shown). The specificity of the m-PCR assay was examined by isolating genomic DNA from 20 different *Cp. abortus*, 5 *Cp. pecorum*, and 4 *C. burnetii *strains. The m-PCR specificity was satisfactory as all *Chlamydophila *and *Coxiella *tested strains gave specific PCR product. However no amplification was noted using DNA from any of the other bacterial pathogens suspected to be present into tested clinical samples (data not shown). PCR products obtained from infected clinical samples with *Cp. abortus*, *Cp. pecorum *and *C. burnetii *and from the corresponding reference strains AB7, iB1 and Nine Miles were subsequently digested with *AluI *restriction enzyme. The electrophoresis analysis showed that the generated fragment profiles obtained with both PCR products amplified from infected samples and from the involved bacteria were similar (Figure 3). In addition, we sequenced the amplified DNA products from three clinical samples infected individually with *Cp. abortus, Cp. pecorum*, or *C. burnetii *and found the amplified fragment exactly matched the sequence of the three bacteria (data not shown).

### m-PCR analysis of clinical samples

Purified DNA from a total of 253 biological samples obtained from ruminant herds known to be infected with *Chlamydophila *or *Coxiella *was analyzed by m-PCR. Overall, 67 samples were tested PCR positive for at least one of the three pathogens: 16 (24%) samples (13 vaginal swabs and 3 placentas) were positive for *Cp. abortus*, 2 (3%) samples were positive for *Cp. pecorum *(1 vaginal swab and 1 placenta) and 49 (73%) samples (33 vaginal swabs, 11 raw milks, 4 faeces and 1 placenta) were positive for *C. burnetii*. No simultaneous infection with the three bacteria was observed. However, two vaginal swabs taken from a sheep flock were positive for both *Cp. abortus *and *C. burnetii*. Among the 67 samples tested positive by m-PCR, 42 (63%) were taken from ruminants that aborted while 25 (37%) were collected from animals that lambed normally. In addition, 14 (21%) of the PCR positive ruminants were serologically negative.

### Bacterial isolation

*Chlamydophila *and *Coxiella *isolation attempts were performed on 20 different PCR positive samples to confirm the presence of the involved bacteria. Using blind passages on McCoy monolayer cell culture then in specific pathogen-free eggs, three *Chlamydophila *isolates were obtained successfully from vaginal swabs taken from ewes that aborted. The RFLP-PCR of 16S–23S rRNA intergenic region showed that the three isolates belonged to *Chlamydophila *family including two *Cp*. *abortus *(named ABt5 and Bell2) and one *Cp. pecorum *(named AKt). In addition, the intraperitoneal inoculation of OFI mice then on embryonated hen eggs led to the successful isolation of two characteristic *C. burnetii *strains, CBO7 and CBO8 from vaginal swab and from milk samples of aborted ewes respectively.

## Discussion

Previous studies have reported *C. burnetii *[19] and *Cp. abortus *[20] detection in clinical samples taken from sheep flocks after lambing or abortion. Clinically unapparent intestinal infections caused by *Cp. pecorum *have also been reported to be prevalent in both abortion-affected and unaffected ruminant flocks [1,30]. In addition, a recent study has shown that *Cp. pecorum *was more widespread in cattle than *C. abortus*, and the bacteria were frequently detected in vaginal swabs and faecal samples [31]. Thus, it is necessary to have an approach that can detect and differentiate all relevant organisms using the same sample and the same assay. A highly sensitive real-time PCR method suitable for large-throughput routine detection, quantification, and differentiation of *chlamydophila *DNA from vaginal swab and milk samples was established [32]. In addition, a DNA microarray probe assay, based on highly discriminatory sequences of the 23S rRNA gene, was used for *Chlamydia *and *Chlamydophila *identification and all various species differentiation from clinical samples [33]. The clinical features of abortion caused by *Cp. abortus *and *C. burnetii *are very similar and such mixed infections have been suggested to be a common occurrence in sheep and goat flocks [34]. A duplex real time PCR was developed to simultaneously detect *Cp. abortus *and *C. burnetii *in broad range of abortion products of cattle [22]. However, to our knowledge, this is the first study to test the ability of a multiplex PCR assay to detect and, identify the presence simultaneously of *Cp. abortus, Cp. pecorum *and *C. burnetii *in herds as well as in individual animals.

Preferential amplification of one target sequence over another is a known phenomenon in multiplex PCRs and a loss of sensitivity is often observed when combined a large number of primer sets in a single reaction. In this study, the PCR reaction conditions were carefully optimised and, the ratio of each primer pair was adjusted to obtain maximum sensitivity. Despite the presence of three primer sets in the PCR mixture, the m-PCR was able to detect all tested bacteria at a high level of sensitivity. The amplification experiments performed with both purified genomic DNA of bacteria and with spiked clinical samples allowed to obtain a detection limit of 50 genome copies per PCR reaction which is acceptable for diagnostic use.

Due to the lack of comparative data and, to the absence of a gold standard for the molecular diagnosis of the three pathogens, it was difficult to compare the efficiency of this m-PCR with other PCR methods previously described. However, the data obtained in this study showed that our m-PCR was ten-fold less sensitive than the real-time multiplex-PCR assays already described for Chlamydios and Q fever [31,33,35,22]. The sensitivity of this assay could be further increased by adapting the m-PCR to a real-time multiplex PCR format. Real-time quantitative PCR methods offer an attractive advantage, in the clinical diagnostic laboratory, to detect and quantify multiple pathogens simultaneously. However, the routine and the high-throughput analysis cost remains very high, especially for emerging countries. Attempts to isolate *Chlamydophila *and *Coxiella *strains were performed on 20-different PCR positive samples to confirm the presence of the involved bacteria and to compare the efficacy of the two diagnostic methods as well. All attempts to pathogen isolation were not successful and, only two *Cp*. *abortus*, one *Cp. pecorum *and two *C. burnetii *strains isolates were obtained from vaginal swabs and milk samples. Fifteen m-PCR positive samples were negative upon selective culture suggesting that the m-PCR method detected bacteria that are unable to grow *in vitro*. In our study, the investigated animals were already receiving antibiotic therapy at the time of sampling. When antibiotic treatment compromises the chance of bacterial isolation, PCR detection is not affected by the lack of viability of the microorganism and is more sensitive than culture for the detection of non-viable organisms and cellular DNA that have not been cleared.

The performance of the m-PCR in field studies with infected flocks that reported the occurrence of the two zoonotic diseases further validates its use as an optimal tool for surveillance for chlamydiosis and Q fever. Thus, our investigation showed that these two infections were widespread within the tested flocks as evidenced by the presence of the *Cp. abortus *and *C. burnetii *m-PCR products in over 25% of the tested clinical samples. Two vaginal swab samples were contaminated with both *Cp. abortus *and *C. burnetii *and the ability of the multiplex assay to detect dual infections was therefore known. Recently, an outbreak of enzootic abortion in ovine and caprine herds caused by mixed infections was reported and both *Cp. abortus *and *C. burnetii *were simultaneously detected, using a simplex PCR, in aborted female placentas and foetuses [36]. During our study, the developed m-PCR allowed the detection of *Cp. pecorum *strain in vaginal swab taken from a female ewe that had aborted in one Tunisian flock. The RFLP-PCR analysis of 16S–23S rRNA intergenic region confirmed that the isolated strain belonged to *Cp. pecorum *specie. These data and those reported previously regarding *Cp. pecorum *involvement in abortion in Tunisia and in Morocco (unpublished data) indicated that *Cp. pecorum *may cause abortion in small ruminants in North Africa countries. *Cp. pecorum *pathogenicity may be associated with nutritional deficiency or parasitic infestations as are often encountered in theses countries. It could be also considered that no pathogenic *Cp. pecorum *strains might be spread from the intestine through the blood circulation because of some unknown physiopathologic events and reach the placenta where they induce abortion. The recent finding that mixed infection with *Cp. abortus *and *Cp. pecorum *was associated with abortion in water buffalo cows in the southern of Italy [37] suggests that *Cp. pecorum *could also be involved in abortion in large ruminants. Nevertheless, it is still unknown whether or not *Cp. pecorum*-related abortion might be either a consequence of *Cp. pecorum *alone or an enhancement of its pathogenesis mediated by the co-infection with *Cp. abortus*.

## Conclusion

The m-PCR assay developed in this study provides a new tool for Chlamydiosis and Q fever diagnosis. The usefulness of this assay to detect the animals that actively shed the bacteria may prevent animal, human, and environment contamination. In addition, since *Cp. pecorum *infection is still not well understood, this m-PCR may yield new insights into the pathogenesis of Chlamydiosis disease.

## Authors' contributions

MB conceived, designed, and coordinated the study, carried out all the molecular and PCR studies, analyzed and interpreted all results, and drafted the manuscript. AbR performed the animals sampling, the ELISA immunoassay, and the bacteria isolation. KSB participated in the bacteria isolation and characterization as well as the sequence alignment. AR participated in the study coordination and gave a final approval of the version to be published. All authors read and approved the final manuscript.
